# Assessment of Some Physicochemical Parameters and Heavy Metals in Hand-Dug Well Water Samples of Kafta Humera Woreda, Tigray, Ethiopia

**DOI:** 10.1155/2021/8867507

**Published:** 2021-02-15

**Authors:** Kiros Gebremichail Gebresilasie, Goitom Gebreyohannes Berhe, Amanual Hadera Tesfay, Samuel Estifanos Gebre

**Affiliations:** ^1^Tsegede Wereda Education Office, Tigrai Regional State Education Bureau, P.O. Box 231, Mekelle, Ethiopia; ^2^College of Natural and Computational Sciences, Mekelle University, P.O. Box 823, Mekelle, Ethiopia

## Abstract

Groundwater is one of the most important sources of drinking water in Kafta Humera Woreda; hence, it is important to assess the quality of these water sources. The aim of this study was to assess the levels of some physicochemical parameters and heavy metals in hand-dug well water sources of Kafta Humera Woreda. The results showed that the physicochemical concentrations of the hand-dug well water samples were given as follows: temperature, 27.67 ± 0.15 to 28.30 ± 0.25°C; pH, 6.90 ± 0.33 to 8.20 ± 0.36; dissolved oxygen, 5.60 ± 0.06 to 6.2 ± 0.04 mg/L; turbidity, 1.67 ± 0.02 to 1.89 ± 0.03 NTU; EC, 148.50 ± 0.89 to 932.00 ± 0.98 *μ*S/cm; TDS, 105.80 ± 0.62 to 664.28 ± 0.70 mg/L; total hardness, 71.80 ± 3.05 to 295.30 ± 2.38 mg/L; total alkalinity, 75 ± 5.0 to 215 ± 5.0 mg/L; calcium, 12.02 ± 0.82 to 75.88 ± 0.93 mg/L; magnesium, 9.80 ± 0.80 to 25.70 ± 0.17 mg/L; potassium, 0.130 ± 0.003 to 0.86 ± 0.04 mg/L; sodium, 2.20 ± 0.16 to 12.75 ± 0.87 mg/L; chloride, 12.86 ± 0.02 to 42.72 ± 0.20 mg/L; sulfate, 17.24 ± 0.96 to 118.67 ± 0.46 mg/L; phosphate, 0.018 ± 0.005 to 0.020 ± 0.002 mg/L; and nitrate, 1.86 ± 0.03 to 5.43 ± 0.06 mg/L. Generally, the concentrations of most physicochemical parameters of the hand-dug well water samples of Kafta Humera Woreda were within the permissible limit of World Health Organization and Ethiopian Standard Agency guideline for drinking water. The statistical Pearson's correlation analysis on the water quality parameters revealed that all parameters are more or less correlated with each other. Electrical conductivity and total dissolved solids of the water samples were found to be significantly correlated with total hardness (*r* = 0.989), total alkalinity (*r* = 0.827), calcium (*r* = 0.988), magnesium (*r* = 0.881), sodium (*r* = 0.995), potassium(*r* = 0.996), chloride (*r* = 0.998), sulfate (*r* = 1), and nitrate ions (*r* = 0.972). Out of the selected seven heavy metals, Fe, Cu, Zn, Mn, Cr, Cd, and Pb, only iron was detected in all water samples and its concentration was above the permissible limit of WHO and ESA for drinking water. Therefore, the government should adopt some treatment technologies such as sedimentation and aeration to minimize the concentration of iron for safe drinking the water to the community of Kafta Humera Woreda.

## 1. Introduction

Water is one of the most important compounds of the ecosystem, and all living organisms on Earth need water for their survival and growth. It is also used for drinking, cooking, agricultural activities, and industrial activities; transportation and recreation are among the most uses of water [1]. Water can be obtained from surface water sources such as freshwater, lakes, rivers, streams, and groundwater sources such as hand-dug well and borehole water. Groundwater is a major source of water for domestic use in both urban and rural areas of the world. It contains over 90% of the freshwater resources and it is an important reserve of good quality water [2]. Groundwater quality has become an important water resources issue due to rapid increase of population, rapid industrialization, unplanned urbanization, and too much use of fertilizers and pesticides in agriculture [3]. Good quality of water is essential for life, but the occurrence of physicochemical parameters and heavy metals above the permissible standards make it unsafe for drinking. The quality of drinking water should be checked at regular time interval because due to the use of contaminated drinking water, human population suffers from a variety of waterborne diseases [1]. The majorities of the populations in developing countries are inadequately supplied with potable water and are thus bound to use water from sources like shallow wells and boreholes that have high potential of contamination and provide the unsafe water for domestic and drinking purposes [4]. The availability and amount of freshwater continue to decrease from time to time in Africa. In Sub-Saharan African countries, the requirements of water for domestic and industrial purposes are usually not met. The natural chemical quality of groundwater is generally good, but at higher concentration a number of constituents can cause problems for water use [5].

In Ethiopia, the most common source of drinking water used to supply major urban and rural communities is obtained from hand-dug wells, boreholes, and shallow wells and covers about 85% of the public supply [6]. Hand-dug well water source is the major source of water for drinking and other domestic purposes for the community of Kafta Humera community (Kunama Adigoshu, Hagere selam, May Woyni, and Habesha Adigoshu. However, the physicochemical parameter and heavy metal concentrations of the hand-dug well water sources have not been tested before in the study area and to compare the results with the minimum requirement of World Health Organization (WHO) and the Ethiopian Standard Agency (ESA). Therefore, the aim of this study is to assess the levels of some physicochemical parameter and heavy metal concentrations of hand-dug well water samples of Kafta Humera Woreda, Tigray, Ethiopia.

### 1.1. Description of the Study Area

**Figure 1:**
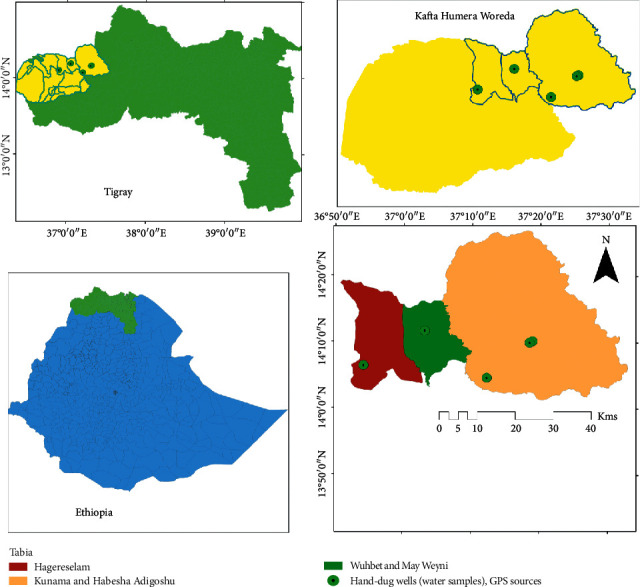
Map of the study area.

Water samples were collected from Western Zone of Tigray Region, Kafta Humera Woreda specifically Kunama Adigoshu, Hagere Selam, May Woyni, and Habesha Adigoshu Kebelles (Figure 1). It is located 587 km Northwest of Mekelle, the capital city of Tigray Region, bordering Sudan on the West and Eritrea on the North. Geographically, the area lies between 13° 40′ N and 14° 27′ N and between 36° 27′ E and 37° 32′ E with an elevation varying from 550 m to 2800 m above sea level. The annual rainfall in the area ranges from 500 to 1500 mm. The relative humidity is highest during the months of rain (June-July) and lowest at the end of the dry season in April [7]. Based on the 2007 national census conducted by the central statistical agency of Ethiopia, the Woreda has a total population of 92,167 [8].

## 2. Materials and Methods

### 2.1. Sampling and Preservation

Water samples were collected from four sampling sites Kafta Humera Woreda (Kunama Adigoshu, Hagreselam, May Woyni, and Habesha Adigoshu) on May 2019. Simple random sampling method was employed on selecting the four hand-dug wells' water from each site of the study area. Water samples were collected in 2 L polyethylene plastic bottle previously washed with detergents, acidified with 5% HNO_3_ for 24 h, rinsed twice with distilled water, and then finally rinsed with sample water three times before filling the sample water [9]. Samples from the wells were obtained by direct immersion of plastic containers into the reservoir handled by 10 m rope and the fetched water was poured immediately into the polyethylene plastic sample containers. Finally, all the collected samples from the study area were labeled, preserved in ice box at temperature of 4 to 10°C to avoid any contamination, and transported to Mekelle University, College of Natural and Computational Science, School of Geochemistry laboratory and Ezana Mining Analytical Laboratory Development P.L.C for analysis. Standard methods [10] were used for sample collection, handling, and preservation to ensure data quality and consistency.

### 2.2. Analysis of Physicochemical Parameters in the Water Samples

The water samples were analyzed for physicochemical parameters such as temperature, pH, turbidity, dissolved oxygen (DO), electrical conductivity (EC), total dissolved solids (TDS), total alkalinity, total hardness, calcium, magnesium, sodium, potassium, chlorine, sulfate, phosphate, and nitrate using standard analytical methods [11]. The pH and total dissolved solids were measured using pH meter (HI-99130, Italy). Electrical conductivity was measured using conductivity meter (JENWAY, multi-3410, UK). Turbidity was measured using turbidimeter (AL 250T-IR, Germany). The temperature of the water samples was measured on-site using mercury thermometer. Dissolved oxygen was determined using multi parameter (Multi-3410, Germany). The total alkalinity was determined using titrimetric method. Total hardness, calcium, and magnesium hardness were determined using EDTA titrimetric method. Chloride concentration was determined using argentometric titration method. The cations potassium and sodium were determined using flame photometry (JENWAY, PFP7, UK). The anions sulfate, phosphate, and nitrate were measured using UV-visible spectrophotometer (Lambda, CE1021, and Australia) [11].

### 2.3. Analysis of Heavy Metal in the Water Samples

Exactly 50 mL of filtered aliquot water sample was pipetted into a digestion flask and mixed with 10 mL of 68% HNO_3_ and then allowed to heat on hot plate for about six hours at a temperature of 80°C until the volume was reduced to one-fifth of its original volume. The solution was cooled, filtered using Whatman number 41 filter paper, and then diluted to 100 mL volumetric flask using distilled water. The concentrations of heavy metals were determined using FAAS (AA240FC, Australia) at the analytical laboratory of Ezana Mining Development P.L.C, Mekelle, Ethiopia [12].

### 2.4. Data Analysis

The physicochemical parameters and heavy metals were compared with WHO and Ethiopian Standard Agency (ESA) guidelines for drinking water. The relationships between the physicochemical parameters and heavy metals in the water samples were established using Pearson product correlation coefficient (*r*) at 1% level of significance. Data collected was statistically analyzed using Statistical Package for Social Sciences (SPSS version 23) [13].

## 3. Results and Discussion

The mean values of different selected physicochemical parameter and heavy metal concentrations are tabulated in Tables 1 and 2.

### 3.1. Temperature

Temperature measures the degree of coldness or hotness of a substance [14]. The temperature of the well water samples varied between 27.67°C and 28.30°C (Table 1). Similar studies conducted in selected Hand-Dug Wells for Water Quality in Ilesa Metropolis, Southwest Nigeria, scored temperature value of 28.04 ± 0.12°C [16]. The values of temperature for all well water samples were above the permissible limit of WHO for drinking water, 25°C [14]. These higher water temperatures could be attributed to the environmental temperature as well as other climatic conditions prevailing in the study area at the time of collecting sample. Higher water temperature increases chemical reactions in the aquifer such as weathering of rocks which leads to the release of the chemical contaminants in water and decreases the level of dissolved in aquatic level [17]. Temperature has a negative correlation with dissolved oxygen (*r* = −0.708) and pH (*r* = −0.666), but positive correlation with turbidity (*r* = 0.892), electrical conductivity (*r* = 0.532), and TDS (*r* = 0.532) (Table 3).

### 3.2. Turbidity

Turbidity is a measure of the degree to which the water loses its transparency due to the presence of suspended particles. The turbidity values of the well water samples varied between 1.67 NTU and 1.89 NTU (Table 1). The recorded turbidity values show that all well water samples had values which were within the permissible limit of WHO and ESA for drinking water below 5 NTU [14, 15]. Similarly, the drinking water quality in the State of Perak, Malaysia, indicates that the turbidity of all the samples studied was below the maximum standard limit of 5NTU [18]. The occurrence of suspended soil, sediment, carbon-based substance, inorganic material, and other invisible living things enhances turbidity. Highly turbid water reduces light penetration to the wells, therefore affecting the level of photosynthesis aquatic plants. This increasing in water temperature is due to absorption of sunlight [19]. Turbidity has a significant negative correlation with dissolved oxygen (*r* = −0.867) at *p* < 0.01 (Table 3).

### 3.3. Electrical Conductivity

Conductivity is a measure of the conducting ability of water and it is determined by the presence of ionic species in the water. Electrical conductivity value for all the sampling sites varied from 148.5 to 932 *μ*S/cm (Table 1). The measured EC value indicates that all examined water samples had values which were within the permissible limit of WHO 750 *μ*S/cm and ESA 1500 *μ*S/cm for drinking water except Kunama Adigoshu well water source in WHO [14, 15]. These results are higher in conductivity than the average conductivity value of the drinking water in the State of Perak, Malaysia, which is 102.1 *μ*S/cm [18]. The presence of a slightly high value of electrical conductivity in the water sample shows that contaminations due to dissolve ions are high, because electrical conductivity is directly proportional to the total dissolved solids. This may be due to the soil type and geology of the study area or the wastes entering from the surrounding water sources [20]. Electrical conductivity shows significant correlation with ten parameters such as sulfate ion and TDS (*r* = 1), total hardness (*r* = 0.989), total alkalinity (*r* = 0.827), calcium (*r* = 0.988), magnesium (*r* = 0.881), potassium (*r* = 0.996), sodium (*r* = 0.995), chloride (*r* = 0.998), and nitrate ions (*r* = 0.972) at *p* < 0.01. This shows that the presence of these ions in dissolved form in water can increase the electrical conductivity of water (Table 3).

### 3.4. Total Dissolved Solids

Total dissolved solids are the amount of mobile charged ions, including minerals, salts, or dissolved metals in a given volume of water in mg/L. The mean total dissolved solids (TDS) obtained from well water samples were from 105.80 mg/L to 664.30 mg/L (Table 1). All the measured well water samples recorded TDS concentration values within the permissible limit of WHO (500 mg/L) and ESA (1000 mg/L) for drinking water, except Kunama well water source in WHO [14, 15]. These results are in agreement with a study conducted in India; total dissolved solids values ranged from 146 mg/L to 467 mg/L [21]. High level of TDS in groundwater may be due to dissolution of weathered materials from rock formation [22]. The occurrence of high values of TDS in certain well water samples could be unpleasant to consumers and may cause excessive scaling in water heaters, boilers, and household equipment [23]. Total dissolved solids show significant positive correlation with ten parameters such as sulfate ion and EC (*r* = 1), total hardness (*r* = 0.989), total alkalinity (*r* = 0.827), calcium (*r* = 0.988), magnesium (*r* = 0.881), potassium (*r* = 0.996), sodium (*r* = 0.995), chloride (*r* = 0.998), and nitrate ions (*r* = 0.972) at *p* < 0.01. This shows that the presence of these ions in dissolved form in water can increase the amount of total dissolved solids in water (Table 3).

### 3.5. pH

pH values of the well water samples varied between 6.9 ± 0.32 and 8.2 ± 0 36 (Table 1). All the wells recorded pH values within the acceptable limit of WHO and ESA standards 6.5–8.5 [14, 15]. Similar results were recorded from groundwater sources of Dire Dawa City, Eastern Ethiopia, which were within the permissible pH limits (6.5–8.5) [24]. The pH has a negative significant correlation with turbidity (*r* = -0.848), but strong positive correlation with dissolved oxygen (*r* = 0.869) value at *p* < 0.01 (Table 3).

### 3.6. Dissolved Oxygen

Dissolved oxygen is an important water quality parameter and it is an indicator of water contamination. The small amount of dissolved oxygen in water indicates the microbial contamination or corrosion of chemical substances in the aquifer [25]. The dissolved oxygen values of the well water samples varied between 5.6 mg/L and 6.2 mg/L (Table 1). From the analyzed result, all well water samples recorded dissolved oxygen values within the acceptable limit of WHO standard for drinking water, 5–7 mg/L [14]. These results are similar to a study conducted in groundwater around Singrauli Coalfield Areas, Singrauli district of Madhya Pradesh (India); it varies from 4.98 to 5.72 mg/L [21]. The level of dissolved oxygen in natural water highly reduced with increasing of water temperature and high organic concentrations as a result of increased decomposer activities [26].

### 3.7. Total Alkalinity

Alkalinity is a measure of the ability of water to neutralize acids and it mainly occurs due to the presence of carbonates and bicarbonates in the water^.^ The concentrations of total alkalinity in the well water samples varied between 75 mg/L and 215 mg/L (Table 1). The measured values of total alkalinity in all well water samples were above the permissible limit of WHO (120 mg/L) for drinking water, except Hagere Selam well water sample [14]. However, the total alkalinity of all well water samples was within the permissible limit of ESA 200 mg/L [15]. These results are in line with a study conducted in Greece where water alkalinity varied from 23.56 to 267.00 mg/L [27]. Total alkalinity has a positive significant correlation with calcium (*r* = 0.78), magnesium (*r* = 0.887), sodium (*r* = 0.813), potassium (*r* = 0.829), chloride (*r* = 0.805), and sulfate(*r* = 0.838) at *p* < 0.01. This indicates that the presence of these ions in dissolved form in water can increase the amount of total alkalinity of water (Table 3).

### 3.8. Total Hardness

Hardness of water is caused by the presence of dissolved polyvalent metallic ions, predominantly calcium ions (Ca^2+^) and magnesium ions (Mg^2+^). The values of total hardness concentrations recorded in the well water samples varied between 71.80 mg/L and 295.33 mg/L (Table 1). These amounts of total hardness concentrations are within the maximum allowable limit of WHO and ESA for drinking water 300 mg/L [14, 15]. Higher water hardness concentration in groundwater may be due to the dissolution rocks containing gypsum and dolomite which are responsible for water hardness [27]. Similar results were observed in Guya Village and its surrounding area, Tigray, Northern Ethiopia; hardness values varied from 170.00 to 327.50 mg/L [28]. Hardness has a significant positive correlation with calcium (*r* = 0.978), magnesium (*r* = 0.939), sodium (*r* = 0.985), potassium (*r* = 0.984), chloride ion (*r* = 0.981), sulfate ion (*r* = 0.992), and nitrate ion (*r* = 0.937) at *p* < 0.01 (Table 3). This implies that the presence of these ions in water may have a direct effect on hardness values. The hardness observed in the hand-dug well water samples of the study area may be permanent hardness, because it has a strong positive correlation with permanent hardness causing ions such as chloride, sulfate, and nitrate salts.

### 3.9. Calcium and Magnesium Ion

Calcium and magnesium ions are the major constituents of various types of rock and they are the most common constituents present in natural waters ranging from zero to several hundred mg/L [29]. The highest calcium amount (75.88 mg/L) of the well water was recorded at Kunama Adigoshu whereas the lowest value (12.02 mg/L) was found in Hagere Selam well water sample (Table 1). All the wells recorded calcium ions within the acceptable limit of WHO and ESA standard for drinking water, 100 mg/L [14, 15]. Calcium ion (Ca^2+^) can occur naturally in groundwater through the dissolution of carbonate minerals and the decomposition of the sulfate, phosphate, and silicate minerals. High calcium concentrations in water may lead to the formation of solid scales in pipes and kitchen utensils and increased soap consumption [30]. The concentration of magnesium ions in the wells varied between 9.80 mg/L and 25.70 mg/L (Table 1). From the analyzed result, all the well water samples recorded magnesium ions within the permissible limit of WHO and ESA constants, 50 mg/L, for drinking water [14, 15]. The main sources of magnesium in the underground water sampled may be attributed to geological sources such as dolomite and other magnesium containing compounds in sediments and soils of the water samples [18]. Calcium and magnesium have a significant positive correlation with potassium, sodium, chloride, sulfate, and nitrate ions (Table 3).

### 3.10. Sodium and Potassium Ion

The natural source of sodium and potassium in groundwater was from the weathering of rocks, but the elevated quantities in contaminated water may be attributed to the release of waste water [23]. The amount of sodium ions in well water samples varied between 2.20 mg/L and 12.75 mg/L (Table 1). The amount of sodium recorded in all well water samples was below the maximum permissible limit of WHO and ESA for drinking water, 200 mg/L [14, 15]. The potassium levels recorded in the study areas were very low ranging from 0.13 mg/L to 0.86 mg/L (Table 1). The concentration of potassium obtained from all well water samples was far below the maximum permissible limit of WHO and ESA constant, 10 mg/L, for drinking water [14, 15]. Similar results were recorded in the groundwater of Dire Dawa City, Eastern Ethiopia, concentration of sodium (7.31 mg/L–74.94 mg/L) and potassium (0.55 mg/L–3.33 mg/L) [24]. Sodium shows a significant positive relationship with chloride (*r* = 0. 994), sulfate (*r* = 0.996) and nitrate (*r* = 0.968) values. Potassium value has a positive strong correlation with sodium (*r* = 0.987), chloride ion (*r* = 0.996), sulfate (*r* = 0.995), and nitrate ion (*r* = 0.971) at *p* < 0.01.

### 3.11. Chloride Ion

Chlorides enter into surface and groundwater from both anthropogenic and natural sources such as from run-off wastes from human activities, discharges of wastewaters into water bodies, use of inorganic fertilizers, landfill leachates, and septic tank effluents [31]. The concentration of chloride ion in the well water samples varied between 12.86 mg/L and 42.72 mg/L (Table 1). The level of chloride ion recorded in all water samples was within permissible limit of WHO and ESA for drinking water, 250 mg/L [14, 15]. These slight variations in the concentrations might be due to different levels of chloride salts in the soil and sediments or a result of differences in degree of wastes entering into the hand-dug wells [32]. The findings of this study are similar to hand-dug wells in Hantebet Catchment, Tigray, and Northern Ethiopia, varying from 15.45 to 49.44 mg/L [33]. Chloride value has a positive strong correlation with sulfate ion (*r* = 0.997) and nitrate ion (*r* = 0.982). This shows that other factors governing the loading of these ions may directly affect chloride distribution at *p* < 0.01.

### 3.12. Sulfate, Phosphate, and Nitrate Ions

The concentration of sulfate ion in the well water samples varied between 17.24 mg/L and 118.67 mg/L (Table 1). From the analyzed result, the sulfate level of all well water samples was within the permissible limit of WHO and ESA for drinking water 250 mg/L [14, 15]. The high sulfate content of some well water samples of the study area may be due to the presence of sulfate containing minerals such as gypsum (CaSO_4_·2H_2_O) and anhydride (CaSO_4_) in the sites [34]. Sulfate has a positive strong correlation value with nitrate (*r* = 0.966) at *p* < 0.01. The phosphate levels obtained in the well water samples of the sampling area were very low, ranging from 0.018 to 0.073 mg/L (Table 1). The concentrations of phosphate in all well water samples were within the permissible limit of WHO and ESA for drinking water, 2 mg/L [14, 15]. The low concentration of the phosphate in the well water samples might be due to the geology of the area [32]. The nitrate levels in the well water samples ranged from 1.86 to 5.43 mg/L (Table 1). All the well water samples recorded nitrate ion concentration within the permissible limit of WHO and ESA for drinking water, 45 mg/L and 50 mg/L, respectively [14, 15]. Nitrate level in groundwater may be attributed to the introduction of sewages of humans and animals from the surrounding of the well, because it is very close to human residents. Sulfate has a significant positive correlation with nitrate value (*r* = 0.966) at *p* < 0.01.

### 3.13. Iron

Iron was the only heavy metal detected in all well water samples of the study area. The concentration of iron measured in all well water samples varied from 0.33 mg/L to 1.86 mg/L (Table 2). From the analyzed result, all the well water samples had iron concentration above the permissible limit of WHO and ESA for drinking water, 0.3 mg/L [14, 15]. The higher value of iron from the study areas could also be from the natural sources. This may be due to the weathering of minerals, soil type, and sediments which are iron-rich materials [32]. Iron may not pose any health hazards but gives a bitter taste to the water when present in large concentrations. People consuming water sources with high concentration of iron were suffering from taste, color, corrosion of plumbing systems, and liver diseases. However, those exposed to low concentration would be highly susceptible to anemia [34]. A similar study conducted in hand-dug wells of Ilaro and Aiyetoro, Ogun State, Southwestern Nigeria, showed ranges from 0.26 to 1.37 mg/L [35].

### 3.14. Copper, Zinc, Manganese, Cadmium, Lead, and Chromium

The elements of Cu, Zn, Mn, Cd, Pb, and Cr were below the detection limit in all hand-dug well water samples of the study area. Therefore, these elements may not have an immediate threat to the health of individuals in the study area who drink this water.

## 4. Concluding remarks

The aim of this study was to assess the level of some physicochemical parameters and heavy metals of water samples from hand-dug well water sources of Kafta Humera Woreda, specifically, Kunama Adigoshu, Hagere Selam, May Woyni, and Habesha Adigoshu Kebelles, Tigray Region, Ethiopia. The results showed that the concentrations of most physicochemical parameters of the water samples, such as turbidity, pH, total hardness, total alkalinity, calcium, magnesium, potassium, sodium, chloride, sulfate, phosphate, and nitrate ions, were within the permissible limit of the World Health Organization and Ethiopian Standard Agency guideline for drinking water with the exceptions of some physicochemical parameters, such as temperature in all hand-dug wells, total dissolved solids, and electrical conductivity in Kunama Adigoshu hand-dug well water sample which was above the WHO permissible limit, but within the permissible limit of ESA for drinking water. The concentrations of total alkalinity were above the WHO permissible limit in all hand-dug well water samples except in Hagere Selam hand-dug well water source. Out of the selected seven heavy metals, Fe, Cu, Zn, Mn, Cr, Cd, and Pb, only iron (Fe) was detected in all water samples and its concentration was above the permissible limit of WHO and ESA for drinking water. Generally, the water quality of the four hand-dug well water samples was good with the exception of some physicochemical parameters and heavy metals. It is recommended to check the quality of the water sources regularly in the study area. This assessment of some physicochemical and heavy metal quality of water is necessary but not sufficient to determine its suitability for drinking purpose. As such, it is recommended that further research should be a study of other trace heavy metals like mercury, arsenic, and biological parameters to confirm the study reliability.

## Figures and Tables

**Table 1:** The physicochemical parameter concentrations (mean ± SD, *N* = 3) of hand-dug well samples of Kafta Humera Woreda.

Physicochemical parameters (mean ± SD)	Water sources				WHO [14]	ESA [15]
	Kunama Adigoshu	Hagere Selam	May Woyni	Habesha Adigoshu		
Temperature (°C)	27.90 ± 0.10	27.73 ± 0.15	28.30 ± 0.25	27.67 ± 0.15	25	—
Turbidity (NTU)	1.79 ± 0.02	1.71 ± 0.03	1.89 ± 0.03	1.67 ± 0.03	5	5
EC (*μ*S/cm)	932 ± 0.98	148.5 ± 0.89	367 ± 0.36	408.2 ± 0.96	750	1500
TDS (mg/L)	664.28 ± 0.70	105.8 ± 0.62	261.57 ± 0.26	291.03 ± 0.60	500	1000
pH	7.4 ± 0.20	7.6 ± 0.22	6.9 ± 0.32	8.2 ± 0.36	6.5-8.5	6.5-8.5
DO (mg/L)	5.7 ± 0.12	5.98 ± 0.05	5.6 ± 0.06	6.2 ± 0.04	5-7	—
TA (mg/L)	215 ± 5.00	75 ± 5.00	185 ± 5.00	165 ± 5.00	120	200
TH (mg/L)	295.3 ± 2.38	71.8 ± 3.05	142.3 ± 3.42	174.57 ± 3.70	300	300
Calcium	75.88 ± 0.93	12.02 ± 0.82	29.13 ± 1.30	33.45 ± 1.32	100.00	75.00
Magnesium	25.70 ± 0.17	9.80 ± 0.80	16.87 ± 0.09	22.07 ± 0.58	50.00	50.00
Sodium	12.75 ± 0.87	2.20 ± 0.16	4.83 ± 0.07	5.67 ± 0.04	200.00	200.00
Potassium	0.86 ± 0.04	0.13 ± 0.03	0.35 ± 0.05	0.36 ± 0.03	10.00	1.50
Chloride	42.72 ± 0.20	12.87 ± 0.02	24.56 ± 0.17	34.95 ± 0.07	250.00	250.00
Sulfate	118.63 ± 0.46	17.24 ± 0.96	47.13 ± 0.33	52.96 ± 0.27	250.00	250.00
Phosphate	0.018 ± 0.006	0.023 ± 0.002	0.072 ± 0.005	0.073 ± 0.002	2.00	2.00
Nitrate	5.43 ± 0.63	1.86 ± 0.26	2.14 ± 0.04	2.37 ± 0.04	45.00	50.00

WHO: World Health Organization; ESA: Ethiopian Standard Agency; SD: standard deviation; ND: not reported.

**Table 2:** The heavy metal concentrations (mean ± SD, *N* = 3, mg/L) of hand-dug well water sources of Kafta Humera Woreda.

Heavy metals (mean ± SD)	Water sources				WHO [14]	ESA [15]
	Kunama Adigoshu	Hagere Selam	May Woyni	Habesha Adigoshu		
Iron	0.97 ± 0.06	1.86 ± 0.24	0.33 ± 0.02	0.69 ± 0.08	0.3	0.3
Copper	BDL	BDL	BDL	BDL	0.5	2
Zinc	BDL	BDL	BDL	BDL	0.2	5
Manganese	BDL	BDL	BDL	BDL	0.5	0.5
Cadmium	BDL	BDL	BDL	BDL	0.01	0.01
Lead	BDL	BDL	BDL	BDL	0.01	0.01
Chromium	BDL	BDL	BDL	BDL	0.05	0.05

BDL: below detection limit; WHO: World Health Organization; ESA: Ethiopian Standard Agency.

**Table 3:** Pearson's correlation analysis of the physicochemical parameters and heavy metals of hand-dug well water samples of Kafta Humera Woreda.

	*T* ^0^	pH	TU	EC	TDS	TH	TA	Ca^2+^	Mg^2+^	K^+^	Na^+^	Cl^−^	SO_4_^2−^	PO_4_^3−^	NO_3_^−^	DO	Fe
*T* ^0^	1
pH	−0.666 ^ ^*∗*^^	1
TU	0.892 ^*∗*^	−0.848	1
EC	0.532 ^*∗*^	−0.110	0.422	1
TDS	0.532 ^*∗*^	−0.110	0.422	1.00^*∗∗*^	1
TH	0.348	−0.022	0.265	0.989^*∗∗*^	0.989^*∗∗*^	1
TA	0.411	−0.239	0.474	0.827^*∗∗*^	0.827^*∗∗*^	0.862^*∗∗*^	1
Ca^2+^	0.246	−0.045	0.157	0.988^*∗∗*^	0.988^*∗∗*^	0.978^*∗∗*^	0.780^*∗∗*^	1
Mg^2+^	0.255	0.137	0.055	0.881^*∗∗*^	0.881^*∗∗*^	0.939^*∗∗*^	0.887^*∗∗*^	0.871^*∗∗*^	1
K^+^	0.221	−0.10	0.188	0.995^*∗∗*^	0.995^*∗∗*^	0.985^*∗∗*^	0.813^*∗∗*^	0.986^*∗∗*^	0.875^*∗∗*^	1
Na^+^	0.309	−0.126	0.259	0.996^*∗∗*^	0.996^*∗∗*^	0.984^*∗∗*^	0.829^*∗∗*^	0.983^*∗∗*^	0.872^*∗∗*^	0.987^*∗∗*^	1
Cl^−^	0.154	−0.129	0.223	0.998^*∗∗*^	0.998^*∗∗*^	0.981^*∗∗*^	0.805^*∗∗*^	0.987^*∗∗*^	0.857^*∗∗*^	0.996^*∗∗*^	0.994^*∗∗*^	1
SO_4_^2−^	0.186	−0.108	0.223	1.00^*∗∗*^	1.00^*∗∗*^	0.992^*∗∗*^	0.838^*∗∗*^	0.988^*∗∗*^	0.891^*∗∗*^	0.995^*∗∗*^	0.996^*∗∗*^	0.997^*∗∗*^	1
PO_4_^3−^	0.252	0.080	0.124	−0.330	−0.330	−0.220	0.213	−0.362	0.081	−0.343	−0.328	−0.374	−0.307	1
NO_3_^−^	0.104	−0.098	0.152	0.972^*∗∗*^	0.972^*∗∗*^	0.937^*∗∗*^	0.681 ^*∗*^	0.973^*∗∗*^	0.768^*∗∗*^	0.971^*∗∗*^	0.968^*∗∗*^	0.982^*∗∗*^	0.966^*∗∗*^	−0.540	1
DO	−0.708^*∗*^	0.869^*∗∗*^	−0.867^*∗∗*^	−381	−0.380	−0.295	−0.472	−0.299	−0.101	−0.371	−0.403	−0.398	−0.375	0.169	−0.363	1
Fe	−0.565	0.214	−0.492	−0.298	−0.298	−0.374	−0.772^*∗∗*^	−0.240	−0.559	−0.271	−0.309	−0.258	−0.318	−0.740^*∗∗*^	−0.079	−0.03	1

^*∗*^Correlation is significant at the 0.05 level (2-tailed). ^*∗∗*^Correlation is significant at the 0.01 level (2-tailed). *T*^0^: temperature (°C); EC: electrical conductivity; Tu: turbidity; TDS: total dissolved solids; TH: total hardness; TA: total alkalinity; Ca^2+^: calcium ion; Mg^2+^: magnesium ion; Na^+^: sodium ion; K^+^: potassium ion; SO_4_^2−^: sulphate ion; PO_4_^3−^: phosphate ion; NO_3_^−^: nitrate ion; DO: dissolved oxygen; Fe: iron.

## Data Availability

The datasets used and/or analyzed during the current study are available from the corresponding author on reasonable request.
